# Fatty Acid Uptake in Liver Hepatocytes Induces Relocalization and Sequestration of Intracellular Copper

**DOI:** 10.3389/fmolb.2022.863296

**Published:** 2022-04-11

**Authors:** Nathaniel H. O. Harder, Hannah P. Lee, Valerie J. Flood, Jessica A. San Juan, Skyler K. Gillette, Marie C. Heffern

**Affiliations:** Department of Chemistry, University of California, Davis, Davis, CA, United States

**Keywords:** copper, fatty acid metabolism and signaling, metal homeostasis, metabolic disease, homeostasis

## Abstract

Copper is an essential metal micronutrient with biological roles ranging from energy metabolism to cell signaling. Recent studies have shown that copper regulation is altered by fat accumulation in both rodent and cell models with phenotypes consistent with copper deficiency, including the elevated expression of the copper transporter, ATP7B. This study examines the changes in the copper trafficking mechanisms of liver cells exposed to excess fatty acids. Fatty acid uptake was induced in liver hepatocarcinoma cells, HepG2, by treatment with the saturated fatty acid, palmitic acid. Changes in chaperones, transporters, and chelators demonstrate an initial state of copper overload in the cell that over time shifts to a state of copper deficiency. This deficiency is due to sequestration of copper both into the membrane-bound copper protein, hephaestin, and lysosomal units. These changes are independent of changes in copper concentration, supporting perturbations in copper localization at the subcellular level. We hypothesize that fat accumulation triggers an initial copper miscompartmentalization within the cell, due to disruptions in mitochondrial copper balance, which induces a homeostatic response to cytosolic copper overload. This leads the cell to activate copper export and sequestering mechanisms that in turn induces a condition of cytosolic copper deficiency. Taken together, this work provides molecular insights into the previously observed phenotypes in clinical and rodent models linking copper-deficient states to obesity-associated disorders.

## Introduction

Excess consumption of high-calorie diets rich in fats and sugars are linked to adverse physiological effects, such as increased oxidative stress and inflammation (Joshi-Barve et al., 2007; Klevay, 2011; Statovci et al., 2017; Hieronimus et al., 2019; Perdomo et al., 2019). The increased prevalence of such diets is strongly associated with the rise of metabolic diseases including diabetes and non-alcoholic fatty liver disease (NAFLD) (Cusi, 2009; Lomonaco et al., 2011; Softic et al., 2016; Antonucci et al., 2017). The recent literature suggests that excess macronutrient load may result in disruptions in copper trafficking (Park et al., 2015; Heffern et al., 2016; Song et al., 2018; Harder et al., 2020). The essential metal micronutrient copper serves vital roles in signaling and in enzymatic cofactors for key biological processes ranging from mitochondrial respiration to radical scavenging (Lutsenko, 2010; Nevitt et al., 2012; Ackerman and Chang, 2018). However, due to its redox activity, misregulated copper can also be deleterious *via* increased radical formation and DNA damage. Thus, copper must be tightly regulated *via* an intricate network of transporters and chaperones to maintain proper levels and control its localization (Lutsenko, 2010; Bost et al., 2016; Lutsenko, 2016). Copper is introduced into cells *via* membrane importers and then trafficked with dedicated chaperones to their directed targets, including metallothioneins, mitochondrial enzymes, and exporter proteins such as ATP7A and ATP7B (Lutsenko et al., 2007; Lutsenko, 2016; Członkowska et al., 2018). The importance of proper copper homeostasis is evidenced in diseases such as Menkes disease and Wilson’s disease, deadly disorders resulting from mutations in the copper transporters ATP7A and ATP7B, respectively (Cope-Yokoyama et al., 2010; Członkowska et al., 2018; Horn and Wittung-Stafshede, 2021). These diseases show remarkably similar phenotypes to diseases induced by high-fat and high-sugar diets, insinuating a link between copper- and macronutrient-derived metabolic misregulation.

In addition to its central role in energy metabolism, the liver is the primary organ for maintaining copper balance in the body. The majority of dietary copper is trafficked to the liver for storage, distribution, and utilization (Lutsenko, 2010). It is in this organ that the copper-binding serum protein, ceruloplasmin, is synthesized and metalated (Jiang et al., 2016; Linder, 2016; Linder, 2020). In addition, the liver plays a key role in copper excretion *via* biliary export (Lutsenko, 2016; Linder, 2020). Recent research suggests that dietary fats may affect hepatic copper metabolism. In one study, mice fed high-fat diets showed significant decreases in hepatic copper. In another study, researchers found that human hepatocyte (HepG2) cells exposed to a mixture of palmitic and oleic acids displayed decreases in intracellular copper levels akin to the trends seen in the rodent studies (Arciello et al., 2015; Heffern et al., 2016). The prevailing hypothesis is that changes in copper metabolism are attributed to increased levels of the copper transporter, ATP7B, inducing copper efflux from the cell (Arciello et al., 2015; Heffern et al., 2016). Yet, what instigates alterations to ATP7B, the consequence to intracellular copper pathways, and the interplay with fat consumption and energy metabolism is not well understood.

In this work, we scrutinized how fat accumulation impacts molecular pathways of copper metabolism and sequestration. Our goal was to determine associations between ATP7B expression and cellular copper status in response to fatty acid exposure. We assessed time-dependent changes in gene expression, levels, and localization of major copper proteins in HepG2 cells treated with palmitic acid (PA), a 16:0 saturated fatty acid. At early time points of PA exposure, we identified elevations in upstream regulators of the ATP7B export machinery alongside protein changes typically associated with a cellular state of copper overload. This is accompanied by perturbations in mitochondrial health and mitochondrial copper proteins. Prolonged exposure shifts the cellular state to one resembling copper deficiency at later time points, alongside increased expressions of proteins indicative of lysosomal sequestration and membrane localization of copper. From this data, we propose a scheme wherein fat accumulation induces miscompartmentalization of copper to induce a state resembling cytosolic copper overload, activating sequestration and export of copper from the cytosol, leading to a cytosolic state of copper deficiency.

## Materials and Methods

### Cell Culture and Maintenance

Human hepatocyte carcinoma cells (HepG2) were grown in complete DMEM media (31053036, Thermo Fisher Scientific) with 10% Avantor Seradigm premium grade fetal bovine serum (97068-085, VWR), 1 mM sodium pyruvate (11360070, Thermo Fisher Scientific), 100 IU penicillin and 100 μg/ml streptomycin (MT30002CI, Thermo Fisher Scientific), and 2 mM L-glutamine (25-030-081, Gibco). The cells were subcultured every 2–3 days at 70% confluence. The HepG2 cells were gifted to us from Dr. Patricia Oteiza’s laboratory. All experiments were performed on cells between passages 10 and 20. Sterile culturing and assay plates were used for the following experiments. Cells were regularly tested for mycoplasma every 6 months using the MycoAlert assay (LT07-701, Lonza).

### General Procedure for Cell Stimulations

HepG2 cells were stimulated with 250 µM PA solution in MEM media (Cremonini and Oteiza, 2018). The stock PA stimulation solution (stock concentration of 8 mM) was prepared by adding sodium palmitate (P9767, MilliporeSigma) to a 10.5% w/w solution of fatty acid-free bovine serum albumin (BP9704100, Thermo Fisher Scientific) in DMEM (31053036, Thermo Fisher Scientific) with 25 mM HEPES (15630-080, Gibco). The solution was stirred for at least 4 h at 50°C until sodium palmitate was completely dissolved. The solution was filtered using a 0.22-µm cellulose acetate filter (976134, Thermo Fisher Scientific) and diluted to a final concentration of 250 µM in MEM (51200038, Thermo Fisher Scientific) solution with 10% Avantor Seradigm premium grade fetal bovine serum (97068-085, VWR), 1 mM sodium pyruvate (11360070, Thermo Fisher Scientific), 100 IU penicillin and 100 μg/ml streptomycin (MT30002CI, Thermo Fisher Scientific), and 2 mM L-glutamine (25-030-081, Gibco). Solutions of CuCl_2_ were prepared in nanopure water (Millipore) at 200 mM and diluted to 200 µM in the BSA control media. Solutions of bathocuproinedisulfonic acid disodium salt (B1125, MilliporeSigma) were prepared at 20 mM in nanopure water and diluted to 200 µM in BSA control media (10.5% w/w solution of fatty acid-free bovine serum albumin in DMEM and 25 mM HEPES). For each stimulation, HepG2 cells were seeded (see description of experiments below for cell counts) and left to adhere overnight in complete DMEM media. Media were aspirated and cells were then washed once with PBS warmed to 37°C. PBS was aspirated and stimulation media were added to cells, as described for each experiment following.

### Cell Viability and Lipid Staining Assays

Cell viability was assessed using an MTS assay (G3582, Promega) at 24 h. HepG2 cells were seeded in a clear-bottom 96-well plate at 10,000 cells per well and stimulated, as described before. The MTS reagent was added and incubated at 37°C for 1 h before detection of absorbance at 490 nm on a plate reader (SpectraMax i3x, Molecular Devices). Oil O Red was used to assess intracellular fat accumulation within cells plated at 300,000 cells per well in 6-well plates. Cells were stimulated as previously described, washed with PBS, and fixed with 4% paraformaldehyde for 30 min. The Oil O Red stock solution was prepared as 0.5% Oil O Red (NC0961554, Thermo Fisher Scientific) in isopropanol and diluted to 60% in nanopure water fresh for each use. The working Oil O Red solution was filtered before staining for 10 min at room temperature. After staining, cells were washed three times with PBS and then imaged (EVOS Core XL, Thermo Fisher Scientific). To elute dye for quantification, 250 µl of 100% isopropanol was added to stained cells. Cells were then rocked at room temperature for 10 min in isopropanol before transferring 75 µl of the isopropanol solution from each well to a 96-well plate. The absorbance of eluted dye was measured at 540 nm on a plate reader (SpectraMax i3x, Molecular Devices). Statistics were carried out on Prism 9.1 (Graphpad).

### Western Blot Analysis

HepG2 cells were plated at 300,000 cells per well in a 6-well plate. Cells were stimulated as described before and then lysed at 1, 6, 12, and 24 h in a RIPA buffer (150 mM NaCl, 1% NP-40, 0.5% sodium deoxycholate, 0.1% SDS, and 50 mM Tris–Cl pH 7.4) with an EDTA-free protease inhibitor (PIA32955, Thermo Fisher Scientific) and a phosphatase inhibitor (4906845001, MilliporeSigma). Lysates were vortexed on ice for 20 min before being cleared by centrifugation at 15,000 × g at 4°C. Lysates were frozen at −20°C prior to protein quantification using the BCA assay (71285-3, Invitrogen), and 20 µg protein was prepared with 2-mercaptoethanol (1610710, Bio-Rad), PBS (Gibco), and LDS sample buffer (NP0007, Invitrogen), according to the manufacturer’s protocols, without heating and was loaded into a 4%–12% bis–tris 15-well gel (NW04125BOX, Invitrogen) for probing all proteins, except CCS, Mt2A, and SCO2. To probe CCS, Mt2A, and SCO2, protein solutions were run on a 16% tricine gel (EC66952BOX, Invitrogen). In all cases, gels were run in a MES buffer at 100 V for 1 h and transferred on a low fluorescence PVDF membrane (1704274, Bio-Rad) using a Trans-Blot Turbo Transfer System (Bio-Rad). Membranes were blocked with 5% BSA in TBST (9997S, Cell Signaling Technology) for 1 h at room temperature and incubated with primary antibodies overnight at 4°C. Membranes were washed 3 × 5 min with TBST at room temperature and blotted with secondary antibody in 5% dry milk (31FZ82, Grainger) in TBST. Membranes were washed 3 × 5 min with TBST and submerged in Crescendo HRP substrate solution (Millipore) for 5 min prior to imaging on a ChemiDoc MP Imager (Bio-Rad). Primary antibodies used include anti-ATP7B (ab124973, 1:2,000 Abcam), anti-CCS (sc-55561, 1:1,000, Santa Cruz Biotechnology), anti-SCO2 (ab113758, 1:1,000, Abcam), anti-β-actin (mouse IgG, sc-47778, 1:5,000 SCBT or rabbit IgG, 4970S, 1:5,000, Cell Signaling Technology), anti-SLC46A3 (NBP1-85054, 1:1,000, Novus Biologicals), anti-Mt2A (PA5-102549, 1:1,000, Invitrogen), and anti-hephaestin (sc-393701,1:1,000, Santa Cruz Biotechnology). For secondary antibodies, anti-rabbit IgG HRP-conjugated antibody (7074S, 1:2,000, Cell Signaling Technology) was used for SCO2 and ATP7B, anti-mouse IgG HRP-conjugated antibody (7076S, 1:1,000, Cell Signaling Technology) for CCS, and anti-rabbit IgG AlexaFluor 555 (A21428, 1:5,000, Invitrogen) and anti-mouse IgG AlexaFluor 800 (A32789, 1:5,000, Invitrogen) for β-actin. Images were processed using Image Lab software (Bio-Rad). Densitometry analysis was carried out using Image Lab software (Bio-Rad); all analyses were normalized to the baseline condition, BSA at 1 h.

### Gene Expression Analysis

HepG2 cells were plated at 300,000 cells per well in a 6-well plate. Cells were then stimulated as described before, and mRNA was isolated at 1, 6, 12, and 24 h using the RNeasy Plus RNA isolation kit (74136, Qiagen). mRNA was quantified using a QuickDrop spectrophotometer (Molecular Devices), and 1,000 ng was added to iScript Reverse Transcription Supermix (1708841, Bio-Rad). A C1000 thermocycler (Bio-Rad) was used for reverse transcription. A total of 0.2 ng cDNA was loaded into a master mix of amplification primer and iQ SYBR green (1708882, Bio-Rad) before amplification, and gene expression was observation using a CFX Connect Real-Time PCR System (Bio-Rad). Data were processed on Microsoft Excel by the 2^−ΔΔCt^ method using β-actin as the housekeeping gene. TBP (TATA binding protein) was used as a secondary housekeeping gene to ensure β-actin was stably expressed over the PA stimulation. Primers for real-time PCR analysis are listed in Supplementary Table S1. Statistics were carried out on Prism 9.1 (Graphpad), while a colocalization analysis was carried out on ImageJ (FIJI) using Coloc 2 (Benedict and Zhang, 2017).

### Ceruloplasmin Activity and Quantification

Media were collected from qPCR and western blot stimulations for analysis of ceruloplasmin activity and concentration. Collected media were spun at 500 × g for 10 min at room temperature prior to being aliquoted and frozen at −20°C. Undiluted media were assessed for ceruloplasmin concentration by ELISA (EC4201-1, Assaypro), while media for ceruloplasmin ferroxidase activity was diluted to 1:3 in the assay buffer for colorimetric quantification of activity (EIACPLC, Invitrogen). All experiments were run in four independent biological replicates. Statistics were carried out on Prism 9.1 (Graphpad).

### Immunofluorescence Imaging

HepG2 cells were plated at 150,000 cells per well on acid-washed and sterilized glass coverslips in a 12-well plate. Cells were stimulated as described before and then washed at 1, 6, 12, and 24 h in cold PBS (Gibco) and fixed for 10 min in 4% paraformaldehyde (AAJ19943K2, Thermo Fisher Scientific). Cells were blocked in 10% BSA and permeabilized with Triton X-100. 1:600 anti-ATP7B (ab124973, Abcam) and 1:600 anti-TGN46 (GTX74290, GeneTex) antibodies were used to stain the copper transporter and trans-Golgi network marker, respectively, and anti-rabbit IgG AlexaFluor 488 (R37116, Invitrogen) and anti-sheep IgG AlexaFluor 647 (A21448, Invitrogen) were used as secondary antibodies to fluorescently label the proteins. A DAPI dye (R37606, Invitrogen) was used to stain the nucleus, and coverslips were sealed to glass slides along with Prolong Gold Antifade Mountant (P36930, Invitrogen). Fixed and mounted cells were imaged using a laser scanning confocal microscope (Olympus FluoView FV1000) using a ×60 oil immersion lens at the UC Davis Molecular and Cellular Biology Light Microscopy Imaging Facility.

### Glutathione Oxidation

Oxidized and total glutathione levels were assessed by using the GSH-Glo glutathione assay (V6611, Promega). HepG2 cells were plated at 10,000 cells per well in white 96-well plates. Cells were stimulated for 1, 6, 12, and 24 h before addition of glutathione detection reagents. Oxidized and total glutathione levels were assessed by the bioluminescent signal on the i3x plate reader (Molecular Devices) with an integration of 1,000 ms. Data is expressed as the ratio of oxidized glutathione over total glutathione. Statistics were carried out on Prism 9.1 (Graphpad).

### Mitochondrial Membrane Potential

Mitochondrial membrane potential was assessed by using the JC-1 probe assay (ab113850, Abcam). HepG2 cells were plated at 10,000 cells per well in black, clear-bottom 96-well plates. Cells were stimulated for 1, 6, 12, and 24 h before addition of 1 mM JC-1 solution in the corresponding stimulation media. Cells were incubated for 10 min in the dark at 37°C and washed twice with PBS. Stained cells were imaged on the i3x Plate reader (Molecular Devices) with 535 nm excitation and 590 nm emission for aggregates and 475 nm excitation and 530 nm emission for monomers. Statistics were carried out on Prism 9.1 (Graphpad).

### Metal Analysis

Metal analysis was performed at the Northwestern University Quantitative Bio-element Imaging Center generously supported by NASA Ames Research Center NNA06CB93G. Total cell pellet and extracellular copper were assessed by inductively-coupled plasma mass spectrometry (Thermo Fisher Scientific iCap Qc ICP-MS). Cells were seeded at 300,000 cells per well and stimulated for 1, 6, 12, and 24 h. Media were collected and spun at 500 × g for 10 min. A total of 250 µL media were added to pre-weighed metal-free 15-ml conical tubes (89049-170, VWR), and 250 µl analytical grade 70% nitric acid (A509P500, Thermo Fisher Scientific) was added to the mixture and left at room temperature for 24 h. Cells were washed two times with cold PBS and digested in 250 µl analytical grade 70% nitric acid. After 24 h of acid digestion, 225 µl of cell lysate was transferred to pre-weighed metal-free 15-ml conical tubes. All samples were diluted to 5 ml with 3% analytical grade nitric acid. Copper and phosphorus were assessed by ICP-MS. Intracellular copper concentration is expressed as the ratio of concentrations of copper over phosphorus, while copper concentration in media samples is expressed as the concentration of copper over the mass of the sample.

## Results

### Palmitic Acid Alters Copper Transporter Levels With Time Dependence Prompting Copper Export Mechanisms

**FIGURE 1:**
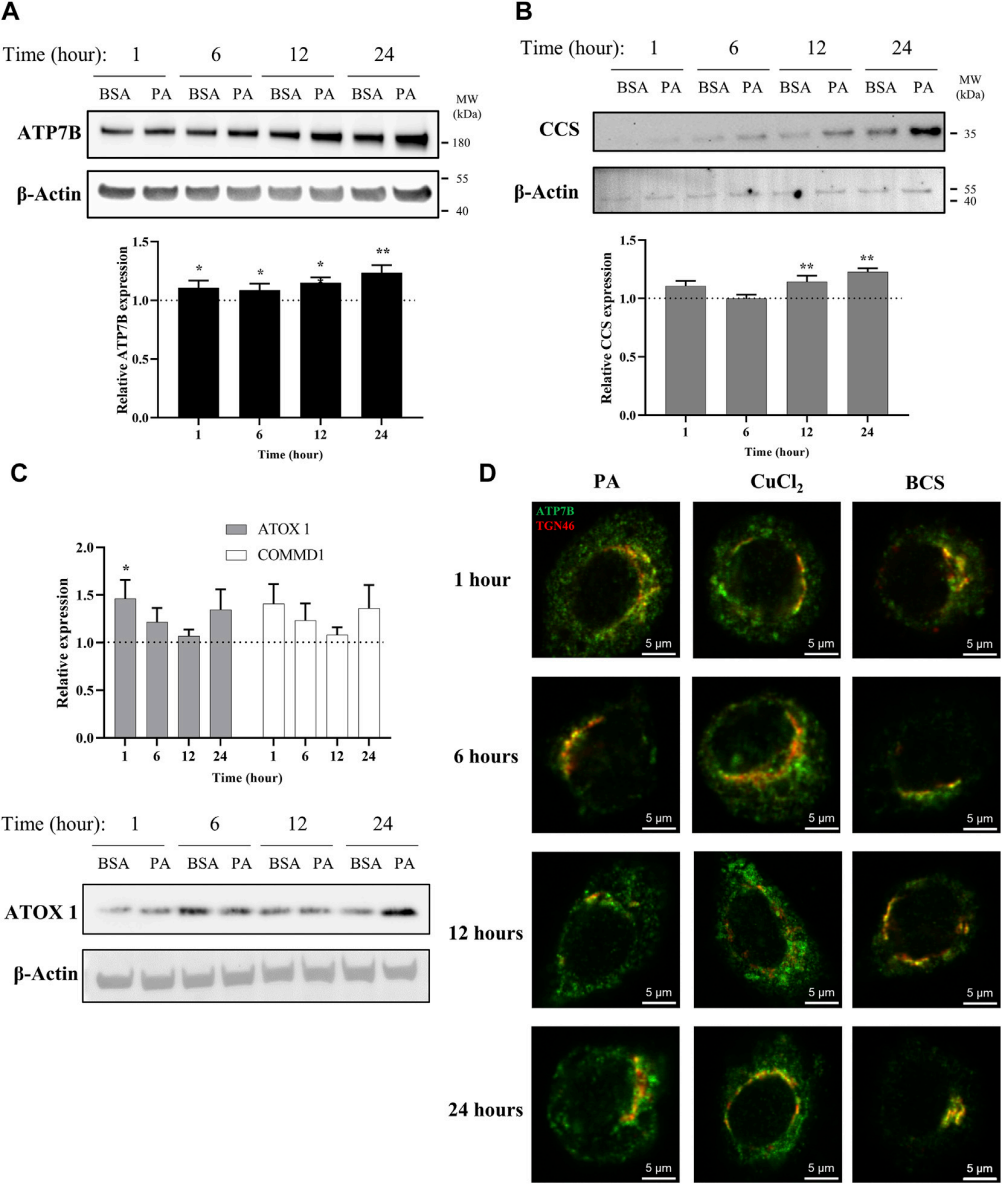
PA induces changes in proteins associated with copper export and induces a subcellular localization of ATP7B that resembles cytosolic copper overload in liver cells. Representative western blot images, densitometry, and gene expression analysis of **(A)** ATP7B (*n* = 10), **(B)** CCS (*n* = 9), **(C)** ATOX1 (*n* = 10), and COMMD1 (*n* = 10) from lysates collected at different stimulation times with PA. Densitometry is shown in Supplementary Figures 1A–C. Gene expression analysis is provided as the levels from PA stimulation relative to BSA stimulation with normalization to β-actin as the housekeeping gene. Mean ± SEM is shown. The Mann–Whitney *U* test was used to assess significance (*p < 0.05, **p < 0.01). **(D)** HepG2 cells were stimulated with PA and 200 μM CuCl_2_ to induce a state of copper overload or 200 μM BCS to induce a state of copper deficiency for 1, 6, 12, and 24 h. Cells were fixed and immunostained, and immunofluorescence imaging was used to capture the subcellular localization of ATP7B (green) and TGN46 (red). Cells were imaged by laser scanning confocal microscopy using a ×60 oil immersion lens. Colocalization is observed by the overlap of signals of ATP7B and TGN46 (yellow).

Previous work showed that high-fat diets perturb hepatic copper metabolism in mice, manifested by an increase in hepatic copper transporter ATP7B (Heffern et al., 2016). Considering the main copper efflux transporter in liver hepatocytes, elevation in ATP7B expression is typically associated with an increased copper export (Lutsenko, 2016; Lutsenko et al., 2007). We first sought to establish whether a hepatocellular model could recapitulate these fat-induced effects on ATP7B expression. HepG2, a human hepatocellular carcinoma cell line, has been extensively used to study metabolic processes at the molecular level; in particular, these cells accumulate fat when treated with fatty acids *via* similar mechanisms as the livers of rodents and human patients given high-fat diets (Joshi-Barve et al., 2007; Arciello et al., 2015; Park et al., 2015; Di Bella et al., 2017). With this precedence, we elected to utilize the well-documented model of treating these cells with sodium palmitate, a sodium salt of PA, for 24 h using bovine serum albumin (BSA) as a carrier and solubilizer (Park et al., 2015; Alsabeeh et al., 2018). Sodium palmitate has been used extensively to model fatty acid exposed livers due to its ability to recapitulate lipid accumulation and steatosis in cells, and as such, it provides a basis to study lipid-induced copper misregulation (Gómez-Lechón et al., 2007; Eynaudi et al., 2021). A 24-h treatment of HepG2 with 250 μM PA indeed resulted in an elevation of ATP7B expression as measured by western blotting (Figure 1A; Supplementary Figure S1A). This change in ATP7B was accompanied by an increase in lipid droplets as measured by Oil O Red staining (Supplementary Figure S2A,B), suggesting that this cell-based model could emulate a similar fat-induced change in copper biology that was previously observed in the mouse models (Heffern et al., 2016). No changes in cell viability were observed under these treatment conditions (Supplementary Figure S3). Comparison of metal content of BSA and PA stimulation media showed no difference in copper levels between the two, ensuring that changes in copper metabolism are due to the fatty acid content of the PA treatment (Supplementary Figure S4).

With a viable model in hand, we applied this system to assess the time-dependent effects of fat accumulation on copper metabolism. We tracked the time-dependent changes in both ATP7B protein and mRNA levels in cell lysates and cellular lipid content at 1, 6, 12, and 24 h of stimulation with 250 µM PA. Both ATP7B protein levels and gene expression (Figure 1A; Supplementary Figure S1A), as revealed by western blotting and qPCR analysis, respectively, were elevated with PA treatment relative to the BSA vehicle at all the time points, with the most pronounced changes observed at 12 and 24 h (*p* = 0.0015 and *p* = 0.000011, respectively, for the relative gene expression analysis). The 12-h point at which notable elevation in ATP7B is observed corresponds to the time frame in which elevated fat accumulation is initially observed by Oil O Red staining (Supplementary Figure S2A,B), suggesting a possible correlation between intracellular fat stores and copper regulation.

To determine how the ATP7B changes may correlate with cellular copper status, we evaluated the protein and gene expression levels of the copper chaperone of superoxide dismutase 1 (CCS). Copper-deficient states induce the upregulation of CCS, allowing the protein to serve as a marker for the cytosolic copper status (Culotta et al., 1997; Bertinato et al., 2003; LassiProhaska and Prohaska, 2012; Brady et al., 2014). A previous work has shown that CCS expression increased in the livers of mice fed with high-fat diets (Heffern et al., 2016). When we stimulated the HepG2 cells with PA, we observed an increase in CCS protein and gene expression levels relative to the vehicle in a similar time-dependent manner to ATP7B, with the most notable increases at 12 and 24 h (*p* = 0.0040 for 12 h and *p* = 0.000041 for 24 h for the relative gene expression analysis) (Figure 1B; Supplementary Figure S1B). This elevation in CCS expression suggests that the PA-induced elevation in ATP7B is accompanied by the onset of a cytosolic copper-deficient state.

To further understand copper trafficking pathways associated with PA addition, we evaluated the changes in two copper chaperones associated with ATP7B, ATOX1, and COMMD1. ATOX1 acts upstream to ATP7B in the copper export machinery and is responsible for loading copper into ATP7B (Shanmugavel and Wittung-Stafshede, 2019; Zaccak et al., 2020). COMMD1, somewhat elusive in terms of its exact function, is linked to ATP7B both as a possible upstream regulator of ATP7B stability and in supporting the bile-dependent export of copper downstream of interactions with ATP7B and independent of ATOX1 (Gitlins, 1988; Fleming et al., 1991; de Bie et al., 2007; Stewart et al., 2019). Upon PA treatment, both ATOX1 and COMMD1 showed changes in gene expression with similar time-dependent trends to one another but at earlier time points than ATP7B, with initial increases at 1 and 6 h relative to the vehicle, followed by a drop in the expression (albeit still modestly elevated above the vehicle) at 12 h which increases at 24 h (Figure 1C; Supplementary Figure S1C). The western blot analysis of ATOX1 revealed similar trends between expression and protein levels, with clear increases in PA-stimulated cells at 1 and 24 h. We noted that for ATP7B, CCS, and ATOX1, BSA treatment alone increased their expression over time (Figures 1A–C; Supplementary Figures S1A–C). Such BSA-dependent changes have not been previously noted in the literature and warrant further exploration beyond this study. Nonetheless, the PA-associated changes occur over this baseline increase with the BSA vehicle. The concomitant changes in these proteins as well as the earlier time points at which they occur support the notion that PA perturbs the copper shuttling machinery upstream of ATP7B.

While classically associated with copper excretion, posttranslational regulation of ATP7B plays a role in altering the cellular localization of the metal as reflected in its subcellular localization, which is modulated by the cellular copper status (Lutsenko et al., 2007; Barnes et al., 2009; Polishchuk et al., 2014). Under copper-deficient conditions, hepatocytic ATP7B localizes to the trans-Golgi network (TGN) where it is presumed to load copper into membrane and serum-derived proteins (Shanmugavel and Wittung-Stafshede, 2019). In contrast, excess cytosolic copper prompts a delocalization of ATP7B from the TGN into dispersed cytosolic vesicles and moves to the cell periphery (Weiss et al., 2008; Barnes et al., 2009; Polishchuk et al., 2014). The delocalization of ATP7B away from the TGN has been associated with the transfer or removal of copper away from the cytosol (Barnes et al., 2009). We thus probed the localization of ATP7B in HepG2 cells treated with PA and compared it to induced copper-overload or copper-deficient conditions, which were achieved by respective treatments with 200 µM CuCl_2_ or the copper chelator, bathocuproine-disulfonate (BCS). The cells were fixed at 1, 6, 12, and 24 h after treatment and stained for immunofluorescence imaging of ATP7B (green) and TGN46 (red) as a marker for the TGN (Figure 1D). As expected, BCS-treated cells exhibited ATP7B localization to the TGN, while ATP7B of CuCl_2_-treated cells showed dispersed localization away from the TGN. When the same experiment was performed in cells treated with PA, we observed that ATP7B initially localizes to the TGN at the 6-h time point, consistent with the copper-deficient state. However, at 12 and 24 h, ATP7B localized to the cell periphery in a distribution akin to that seen in CuCl_2_-treated cells, indicating that the observed increase in ATP7B expression may be pointing toward the removal of copper from the cytosol. The colocalization analysis highlighted the similarities between PA- and CuCl_2_-stimulated cells (Supplementary Figures S5A–D) in decreasing TGN/ATP7B colocalization, particularly at the 12-h time point. The similarity between the two treatments suggests that PA induces a cellular response that is observed under copper-overload conditions, despite expressing markers associated with copper deficiency. This data points to a PA-induced shift in copper metabolism that is distinct from changes altering the overall copper levels of the cell as produced by CuCl_2_ or BCS. Furthermore, the time-dependent changes in ATP7B localization within the cell over the course of the stimulations highlight the plasticity of copper trafficking in the cell in response to fatty acid overload.

### Palmitic Acid Sequesters Copper Into the Membrane and Lysosomes

**FIGURE 2:**
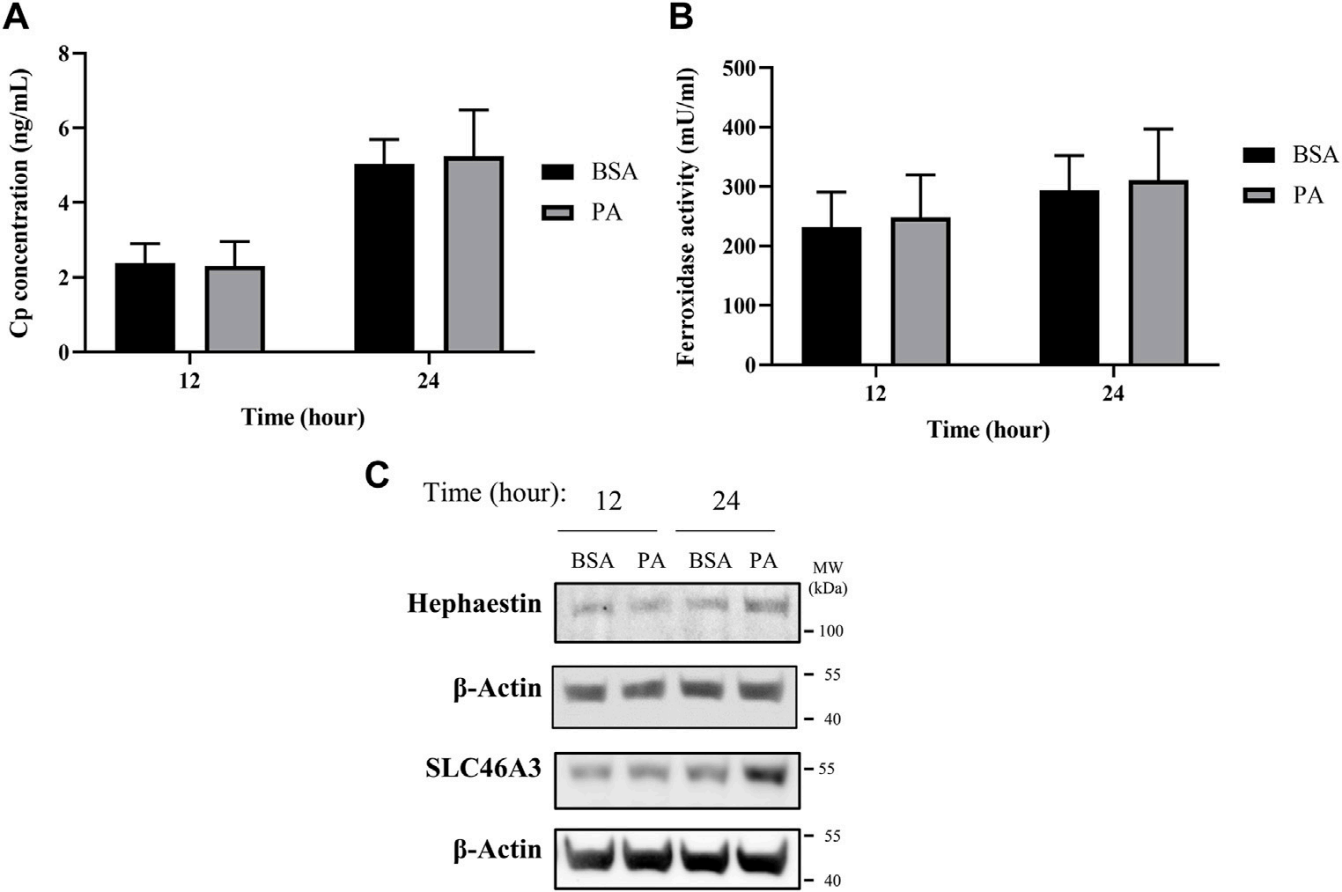
PA does not alter secreted ceruloplasmin (Cp) but induces copper membrane localization and sequestration. **(A)** Cp concentration was measured by ELISA (*n* = 4). **(B)** Copper-dependent ferroxidase activity of Cp in media collected from HepG2 cells treated with PA or BSA for 12 and 24 h (*n* = 4). Mean ± SEM is shown. The Mann–Whitney *U* test was used to assess significance (**p < 0.01). **(C)** Representative western blot images of hephaestin and SLC46A3.

The changes in ATP7B localization point to the ability of PA to induce a removal or sequestration of copper from the cytosol. One of the primary known roles of ATP7B for loading of copper is multi-copper oxidase proteins ceruloplasmin (Cp) and hephaestin (Lutsenko, 2016; Guttmann et al., 2020; Linder, 2020). These homologous multi-copper oxidases are cuproenzymes that contain 6–8 copper atoms per protein. Cp is secreted from the cell and is the most abundant serum copper chaperone, carrying 50%–90% of copper in the blood (Hellman and Gitlin, 2002; Aigner et al., 2008; Linder, 2016). In contrast, hephaestin is anchored to the membrane and presents copper at the cell surface (Vashchenko and MacGillivray, 2013). Both ferroxidases influence iron availability through their copper-dependent ferroxidase activity (Tapryal et al., 2009; Freestone et al., 2016; Jiang et al., 2016). Having observed the PA-induced elevated ATP7B expression and its delocalization from the TGN at 12 and 24 h, we assessed whether these changes were associated with changes in either ferroxidase at these time points. Extracellular Cp concentration and its activity were examined from media taken from the cell supernatant, while cellular hephaestin was assessed by western blotting. No significant differences were observed in either extracellular Cp concentration or copper-dependent extracellular ferroxidase activity upon PA treatment (Figures 2A,B). This is reflected in a similar lack of change in total extracellular copper levels in the media and in total cellular copper levels of whole cell pellets, as measured by ICP-MS (Supplementary Figure S6). In contrast, we observed an increase in the protein levels of hephaestin at 24 h of stimulation with PA (Figure 2C). The concomitant changes in hephaestin, ATP7B, and ATP7B-associated chaperones suggest that PA may induce translocation of copper from the cytosol to the membrane *via* loading into hephaestin, consequently reducing the cytosolic copper content.

Alongside the increases in hephaestin, we investigated other mechanisms involved in the sequestering and redistribution of copper in the cell. Recent research has highlighted SLC46A3 as a protein that is responsible for copper loading into lysosomal units in the liver; as such, we aimed to investigate how SLC46A3 is altered with PA stimulation (Kim et al., 2021). As with hephaestin, PA induces an increase in SLC46A3 protein levels at 24 h of PA stimulation relative to the BSA control (Figure 2C). Thus, in addition to the translocation of copper to the membrane, our data also hints at a PA-induced increase in lysosomal copper sequestration. Taken together, the increase of both hephaestin and SLC46A3 expression at 24 h points to a cellular response to PA involving the mobilization of copper away from the cytosol toward the membrane and lysosomal units.

### Palmitic Acid Stimulation Perturbs Intracellular Copper Balance and Redox Status

**FIGURE 3:**
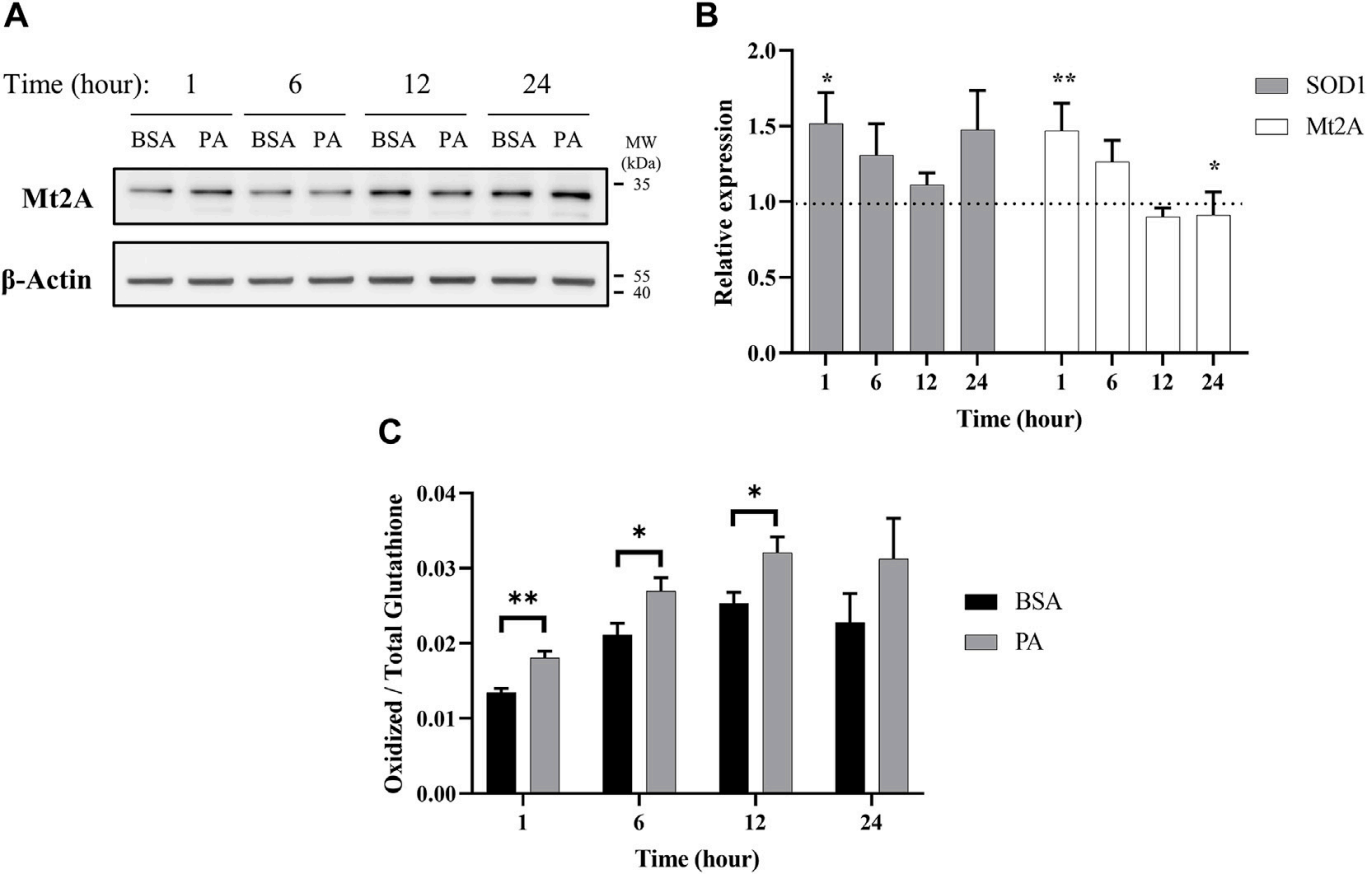
Palmitate stimulations induce changes in cytosolic markers of copper status. Analysis of copper chaperones in the lysates of cells treated with PA or BSA for 1, 6, 12, and 24 h. **(A)** Representative western blot images of Mt2A. **(B)** Gene expression analysis of SOD1 (*n* = 10) and Mt2A (*n* = 10) of PA-stimulated cells relative to BSA-stimulated cells at the same time points (normalized to β-actin as the housekeeping gene). The Mann–Whitney *U* test was used to assess the statistical significance. (*p < 0.05 and **p < 0.01). Mean ± SEM is shown. **(C)** Ratio of oxidized-to-total glutathione in PA-stimulated cells. The unpaired Student’s *t*-test was used to assess significance.

When cytosolic copper levels are perturbed, to counteract oxidative stress and cell damage, the cell utilizes chelators and chaperones to traffic and maintain homeostasis of copper both in terms of overall levels and subcellular distribution (Bertinato et al., 2003; Lutsenko, 2010; Maryon et al., 2013). To this end, we first investigated the effects of PA on metallothionein 2A (Mt2A), a protein implicated in both copper chelation and storage, to establish the cell’s copper buffering dynamics with fatty acid accumulation (Bertinato et al., 2003; Kumar et al., 2013; Heffern et al., 2016; Ostrakhovitch et al., 2016). Mt2A levels are initially elevated upon stimulation of PA (Figures 3A,B), possibly pointing to an initial state of copper overload. This increase subsides with 6 h of PA stimulation and continues to decrease at 12 h, consistent with depression in cytosolic copper levels. By 24 h, we observed a return to Mt2A levels that is similar to those of the control, implying a level of homeostasis. mRNA expression of Mt2A matches the trends observed by western blot wherein there is an initial increase upon stimulation with PA at 1- (*p* = 0.0015) and 6-h treatments, but it decreases upon extended PA exposure with 12- and 24-(*p* = 0.023) h treatments (Figure 3B). As a comparison, we assessed Mt2A gene expression with cells treated with 200 µM CuCl_2_ or 200 µM of the copper chelator BCS. As expected, the addition of CuCl_2_ increases Mt2A expression, whereas BCS decreases Mt2A expression (Supplementary Figure S7). The dynamics of Mt2A suggest that PA treatment initially places the cell’s cytosol in a state of copper overload, prompting the cell to remove copper from the cytosol. This reduction in cytosolic copper then prompts a release of copper from Mt2A to restore copper balance.

Intracellular copper levels are integral to maintaining redox balance in the cytosol. Given the perturbation in cytosolic copper homeostasis by PA, we assessed the consequences of the treatment to the cellular oxidative status. We evaluated the expression of Cu,Zn superoxide dismutase (SOD1), a copper-dependent enzyme responsible for scavenging radical and reactive oxygen species (Culotta et al., 1997). SOD1 gene expression increases at 1 (*p* = 0.023), 6, and 24 h of PA stimulation, corroborating previous research relating copper and oxidative stress in liver fat accumulation (Figure 3B) (Park et al., 2015; Freedman et al., 1989). We also tracked changes in intracellular glutathione oxidative activity. Glutathione is an intracellular peptide that plays a significant role as an intracellular buffer and maintaining the overall reducing environment of the cytosol due to its free thiols and susceptibility to oxidation by radicals (Gallagher et al., 1973; Freedman et al., 1989; Maryon et al., 2013; Ngamchuea et al., 2016). Alongside its redox control, glutathione is responsible for chelating labile metal pools in the cell to reduce Fenton-like chemistry (Thomas et al., 2009). Upon PA addition, we observed an increase in oxidized glutathione at all the time points, with significant increases at 1 (*p* = 0.0072), 6 (*p* = 0.049), and 12 (*p* = 0.043) h (Figure 3C). This may be indicative of either an increase in the overall redox state of the cell, which has been previously observed in fatty acid overload in HepG2 cells, or an increase in the labile copper within the cytoplasm of the cell, or both (Park et al., 2015).

### Palmitic Acid Induces Mitochondrial Dysfunction Linked to Cytosolic Copper Overload

**FIGURE 4:**
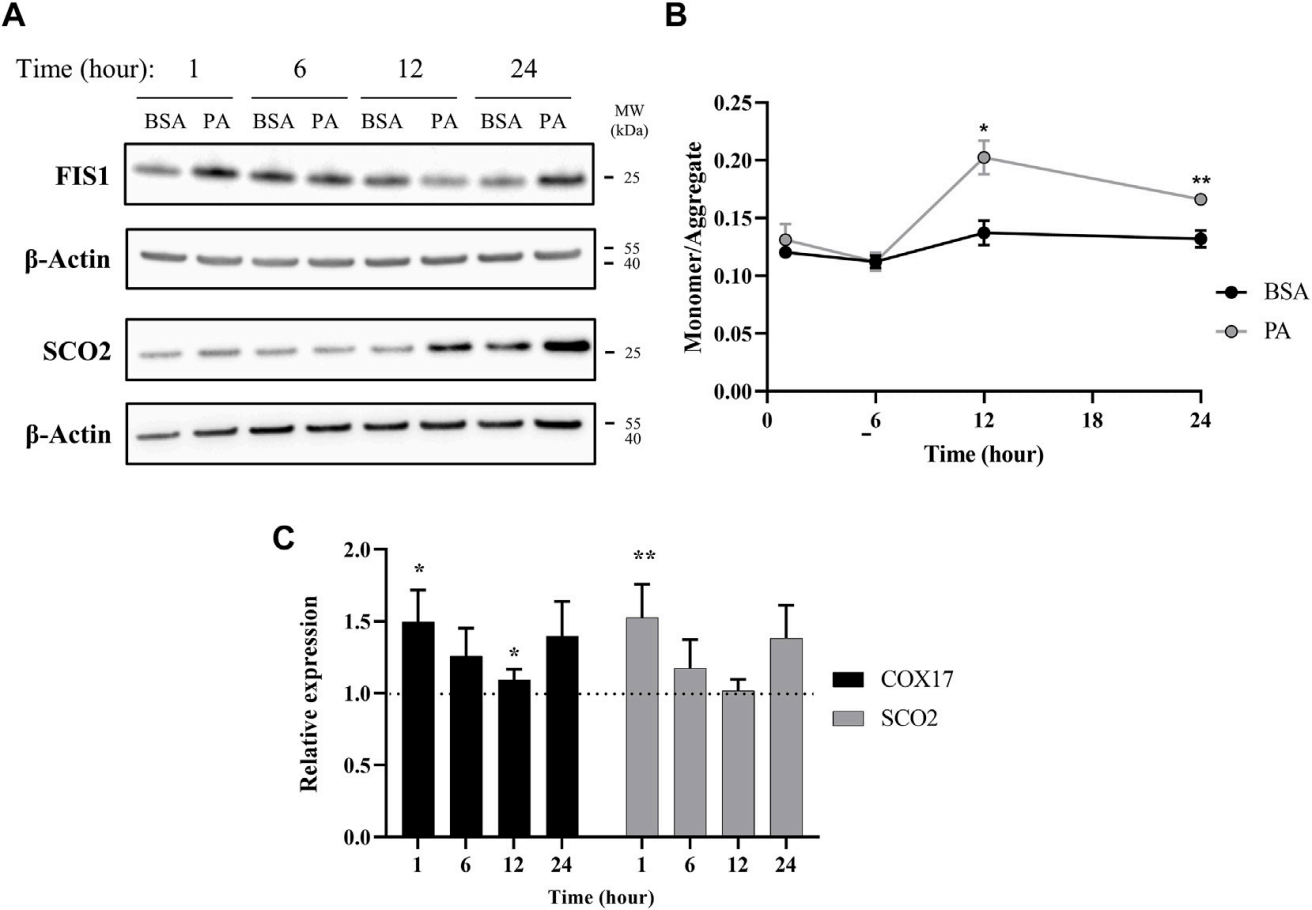
Palmitate stimulations induce changes in mitochondria copper protein regulation. **(A)** Representative western blot images of SCO2 and FIS1 of cells stimulated with BSA or PA. **(B)** Mitochondrial membrane potential was measured using the JC-1 fluorescent dye. Data are shown as monomer/aggregate fluorescence of BSA- and PA-stimulated cells (*n* = 4). Mean ± SEM is shown. The unpaired *t*-test was used to assess significance (*p < 0.05, **p < 0.01). **(C)** Gene expression of COX17 and SCO2 with PA-stimulated HepG2 cells relative to BSA-stimulated cells (*n* = 10) with normalization to β-actin as the housekeeping gene. Mean ± SEM is shown. The Mann–Whitney *U* test was used to assess significance (*p < 0.05, **p < 0.01).

Our data points to an initial state of cytosolic copper overload with PA stimulation that the cell compensates for by removing copper from the cytosol. However, as the PA stimulation solution does not contain exogenous copper, the elevation in cytosolic copper may stem from release of copper from intracellular compartments. Excess fat accumulation is strongly associated with disruptions in proper function of the mitochondria, an organelle that plays a critical role in energy processing from fatty acid oxidation to ATP production (Cobine et al., 2006; Softic et al., 2016; Antonucci et al., 2017; Hill et al., 2017). PA-induced mitochondrial fission, that is, the splitting of the organelle, has been proposed as a main contributor to mitochondrial fragmentation and dysfunction (Sergi et al., 2021). As mitochondria contain high levels of copper as the metal plays critical functions in the electron transport chain, we investigated whether mitochondrial fission occurs at early time points of PA stimulation to potentially release copper in the cytosol (Gallagher et al., 1973; Cobine et al., 2006; Leary et al., 2007). We monitored time-dependent changes in the protein FIS1, a marker for mitochondrial fission, in response to PA stimulation (Koch et al., 2005; Swapna Sasi et al., 2020). FIS1 levels are increased at 1 and 24 h with stimulation of PA (Figure 4A). The early change in FIS1 levels may thus support a mechanism wherein the excess cytosolic copper originates from release of the metal from mitochondrial fission. In support of the changes in mitochondrial fission, we observe changes in mitochondrial membrane potential. This potential was measured by the JC-1 dye, which aggregates in healthy mitochondria and is monomeric in depolarized membranes, is perturbed by the accumulation of fatty acids in the cell as previously reported (Eynaudi et al., 2021). The changes show a slight increase at 1 h that subsides at 6 h but becomes significantly increased at 12 (*p* = 0.0109) and 24 h (*p* = 0.00597), all of which coincides with changes in copper metabolism (Figure 4B). Moreover, PA induces changes in COX17, a copper chaperone that facilitates copper transport to the mitochondria, and SCO2, a chaperone responsible for copper loading into cytochrome c oxidase in the electron transport chain (Nevitt et al., 2012; Polishchuk et al., 2014; Morrell et al., 2020; Cobine et al., 2021). COX17 and SCO2 gene expression are increased in cells stimulated with PA at all the time points (Figure 4C), with notable increases at 1, 6, and 24 h, whereas SCO2 protein levels do not noticeably increase until the 12- and 24-h time points (Figure 4A). Similar increases in SCO2 protein and mRNA levels have been previously observed by Arciello et al. (2015) when HepG2 cells are stimulated with 500 µM of a mixed fatty acid (oleic acid and PA) solution. The degree and direction of changes in these genes at the different time points follow a similar trend as the copper proteins ATOX1, COMMD1, and SOD1 expression, corroborating an overall disruption in copper-associated respiration associated with oxidative imbalance. Taken together, the data supports an initial disruption in copper mitochondrial health that may then induce alterations in the copper subcellular localization.

## Discussion

**FIGURE 5:**
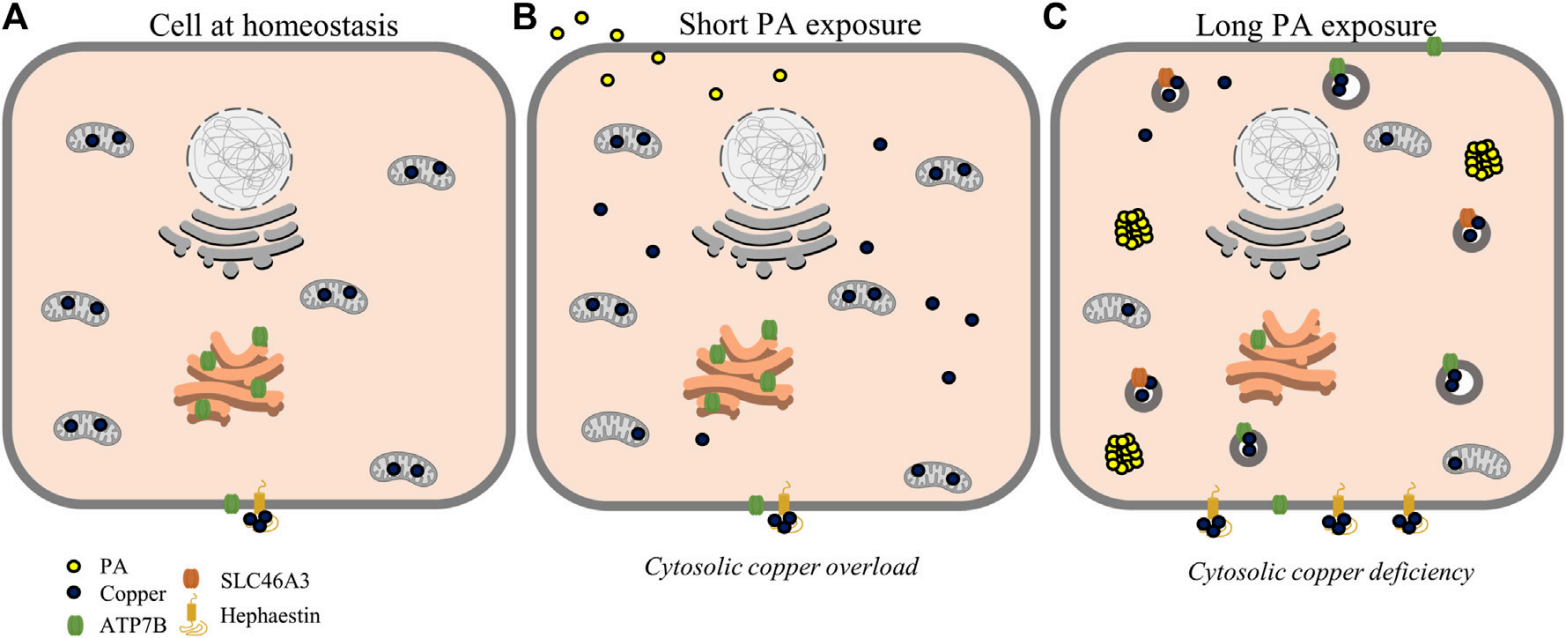
Proposed scheme for possible perturbations of copper homeostasis by PA. At homeostasis **(A)**, copper is mostly sequestered in proteins and organelles with a large concentration in the mitochondria. At short time points of PA exposure **(B)**, cytosolic copper levels are increased alongside mitochondrial dysfunction, leading to a state resembling cytosolic copper overload. With longer PA **(C)**, copper is relocalized toward export by ATP7B and sequestering mechanisms by SLC46A3 and hephaestin resulting in a copper-deficient state.

Emerging studies on copper metabolism are illuminating its vital roles in energy regulation and nutrient processing. In particular, research is revealing homeostatic perturbations in copper metabolism in relation to obesity-related conditions in rodent models and human physiology (Heffern et al., 2016; Harder et al., 2020; Morrell et al., 2020; Lowe et al., 2017). Previous studies have observed increased expression of ATP7B and hepatic copper deficiency in association with hepatic fat accumulation (Arciello et al., 2015; Heffern et al., 2016). In this study, we profiled the changes in copper trafficking that accompany this change in ATP7B using a cellular model, namely, the stimulation of HepG2 cells with PA. Our observations point to a mechanism wherein fatty acids may induce a response akin to cytosolic copper overload that triggers copper transport mechanisms to subsequently generate a copper-deficient-like state. At shorter time points of stimulation, the cell initially demonstrates an increase in copper trafficking and chaperone proteins associated with copper overload, including the elevated expression of proteins involved in the copper export machinery as well an increase in the levels and expression of Mt2A, a putative copper storage protein. This in turn results in a subsequent shift of markers toward a state of copper deficiency, including downregulation and reduced levels of Mt2A, which may indicate the release of copper stores, an increase in protein levels and gene expression of CCS, and an increase in ATP7B. Overexpression of CCS is an established marker of copper-deficient states and is posited to occur as a means for the cell to redirect the limited available copper toward redox balance mechanisms (Freedman et al., 1989; Bertinato et al., 2003; Kumar et al., 2013). Despite the expression of molecular markers of cytosolic copper deficiency at these later time points, the subcellular localization of ATP7B is delocalized from the TGN, a phenotype that is observed in the presence of excess copper (Barnes et al., 2009; Polishchuk et al., 2014). This localization is also accompanied by the increased expression of total ATP7B. While increased ATP7B is typically associated with the activation of copper export, we did not observe changes in total copper concentration of cell pellets nor did we observe increases in extracellular Cp levels or activity. This might suggest that the regulation of ATP7B may instead be functioning toward sequestering or redistributing copper within or at the surface of, rather than exporting the metal from, the cell (Cater et al., 2006; Polishchuk et al., 2014). In support of this hypothesis, we observed an increase in SLC46A3, a protein proposed to play a role in copper loading into lysosomes and potential sequestration (Kim et al., 2021). This membrane protein may be responsible for the changes in cytosolic copper status as copper is redistributed within the cell toward lysosomes. In addition, we observed an increase in protein expression of hephaestin, the membrane-anchored homolog of Cp, supporting a movement of copper to the membrane surface away from the intracellular space (Figure 5) (Vashchenko and MacGillivray, 2013; Jiang et al., 2016; Higuchi et al., 2018). In a mouse model of NAFLD, hepatic hephaestin was significantly increased in mice fed a high-fat and high-cholesterol diet (Higuchi et al., 2018). This increase correlates with our observations in HepG2 cells exposed to PA and highlights the relationship of hephaestin and copper redistribution in nutrient overload. While implications of copper loading into the lysosome and hephaestin are not fully understood and require further investigations, their changes may point to a cellular response to either reduce or utilize cytosolic copper (Vashchenko and MacGillivray, 2013; Bacchi et al., 2014; Polishchuk and Polishchuk, 2016). We noted that COMMD1, which has a posited role in biliary copper export, is altered by PA stimulations (de Bie et al., 2005; Shanmugavel and Wittung-Stafshede, 2019; Stewart et al., 2019); however, as HepG2 cells have altered biliary export mechanisms, our model may be limited in its ability to capture this particular trafficking mechanism (Woolbright et al., 2016).

In further investigating potential mechanisms by which PA perturbs cytosolic copper, we identified alterations in mitochondrial copper proteins. Copper plays a critical role in the electron transport chain and is thus tightly regulated within the mitochondria (Joshi-Barve et al., 2007; Arciello et al., 2015; Ruiz et al., 2021). It has even been proposed that the organelle has the ability to store copper within its matrix (Cobine et al., 2006; Lutsenko, 2010; Nevitt et al., 2012). We observed changes in the gene expression of the mitochondrial copper proteins COX17 and SCO2 with PA addition alongside an increase in the mitochondrial fission protein, FIS1, and mitochondrial membrane depolarization. In addition, the time dependence of the changes of the mitochondrial proteins correlate with the increases in Mt2A, denoting a link between PA-induced effects on mitochondrial health and perturbations to cytosolic copper. Our data agrees with published data wherein changes to SCO2 expression were observed alongside fatty acid treatments in cell culture (Arciello et al., 2015). A hypothesis of SCO2-dependent regulation of copper export from the mitochondria may agree with the observed increases in SCO2 protein and point to a loss of mitochondrial copper (Cobine et al., 2021). Mutations and knockout of SCO2 have been implicated in fatty acid processing and insulin resistance in mouse models, further implicating mitochondrial copper dysfunction in fat accumulation (Hill et al., 2017). Our data complements these findings, as we observed increased glutathione oxidation with the PA treatments, which can relate both to altered copper metabolism as well as changes in the overall redox state of the intracellular environment (Freedman et al., 1989; Maryon et al., 2013). While implications of copper misregulation within the mitochondria are not fully understood, fat overload diseases such as NAFLD have been strongly linked to mitochondrial dysfunction, further connecting such diseases to disruptions in intracellular copper balance (Nassir and Ibdah, 2015; Antonucci et al., 2017). Taken together, our data may point to mitochondrial fission as a potential source for miscompartmentalized copper that triggers subsequent copper shuttling pathways to restore homeostasis. Future studies are required to firmly establish the links between mitochondrial copper regulation and fat accumulation as well as their implications on cell health and disease pathologies.

Diseases that arise alongside fat accumulation are hypothesized to progress in a “multiple-hit” system, with an increase in reactive oxygen species and the accumulation of fat in the liver contributing to disease pathogenesis (Ahmed et al., 2012; Buzzetti et al., 2016; Antonucci et al., 2017). However, the mechanisms behind these phenotypes have yet to be uncovered (Buzzetti et al., 2016; Antonucci et al., 2017; Benedict and Zhang, 2017). As copper can play a role in oxidative stress, the shifts in hepatic copper metabolism that we have observed in response to PA exposure may play a role in the exacerbation of fat-induced cellular stress (Tchounwou et al., 2008; Arnal et al., 2012; Einer et al., 2019). This cytosolic copper overload in turn leads to copper detoxification that appears through export or sequestration as evidenced by increases in SLC46A3 and hephaestin (Barnes et al., 2009; Lutsenko, 2010; Vashchenko and MacGillivray, 2013; Kim et al., 2021). Previous models of copper deficiency demonstrated altered lipid synthesis and metabolism, leading to increased lipid biogenesis and hepatic fat accumulation which could be triggered by the miscompartmenalization of copper observed at later time points (Morrell et al., 2017). This increase in expression of markers of copper deficiency alongside increases in fat accumulation implicates copper misregulation in the process of lipid biogenesis.

From this study, we proposed the following as a mechanism by which copper is misregulated within the liver cell upon PA stimulation (Figure 5). Fatty acid stimulation and subsequent uptake initiate a mislocalization of copper, possibly related to mitochondrial dysfunction. This mislocalization triggers an initial cytosolic response similar to copper overload states, initiating export pathways involving ATOX1, COMMD1, and ATP7B to restore copper balance. This results in sequestration and redistribution of copper, likely to the lysosomes and cell membrane, leading to a state of cytosolic copper deficiency corresponding to increases in CCS levels and decreased levels and expression of Mt2A. This copper deficiency may be exacerbated by export induced by overexpression and relocalization of ATP7B.

In conclusion, our studies reveal mechanistic insights into how the cellular copper distribution of hepatocytes is dynamically perturbed by fat accumulation. We suggested that the shift toward copper export or sequestration is due to cells sensing a state of cytosolic copper overload to restore a level of copper homeostasis. This perceived copper overload activates copper export or sequestering pathways, which subsequently induce a state that resembles copper deficiency. We noted that this study is not an exhaustive study of all the proteins that are potentially involved in copper regulation in the cell and focused primarily on regulatory pathways that were established in the literature. Of note, proteins that warrant a deeper study with regard to their function in hepatic copper trafficking and consequent effects of macronutrients such as fatty acids include but are not limited to ATP7A (which recent studies have highlighted as being expressed in the liver) and CUTC (a protein hinted at playing a role in cytosolic copper balance). Nonetheless, the present study provides a starting point to broaden our mechanistic understanding of how macronutrients such as fat can alter the regulation and compartmentalization of micronutrients such as copper within the cell.

## Data Availability

The original contributions presented in the study are included in the article/Supplementary Material, further inquiries can be directed to the corresponding author.
