# Supplementary material for: Fatty Acid Uptake in Liver Hepatocytes Induces Relocalization and Sequestration of Intracellular Copper
Source: Front Mol Biosci. 2022 Apr 11;9:863296. doi: 10.3389/fmolb.2022.863296 (PMC9036104; doi:10.3389/fmolb.2022.863296)
Supplement: Supplementary file 1 [file DataSheet1.pdf]

Supplementary Material

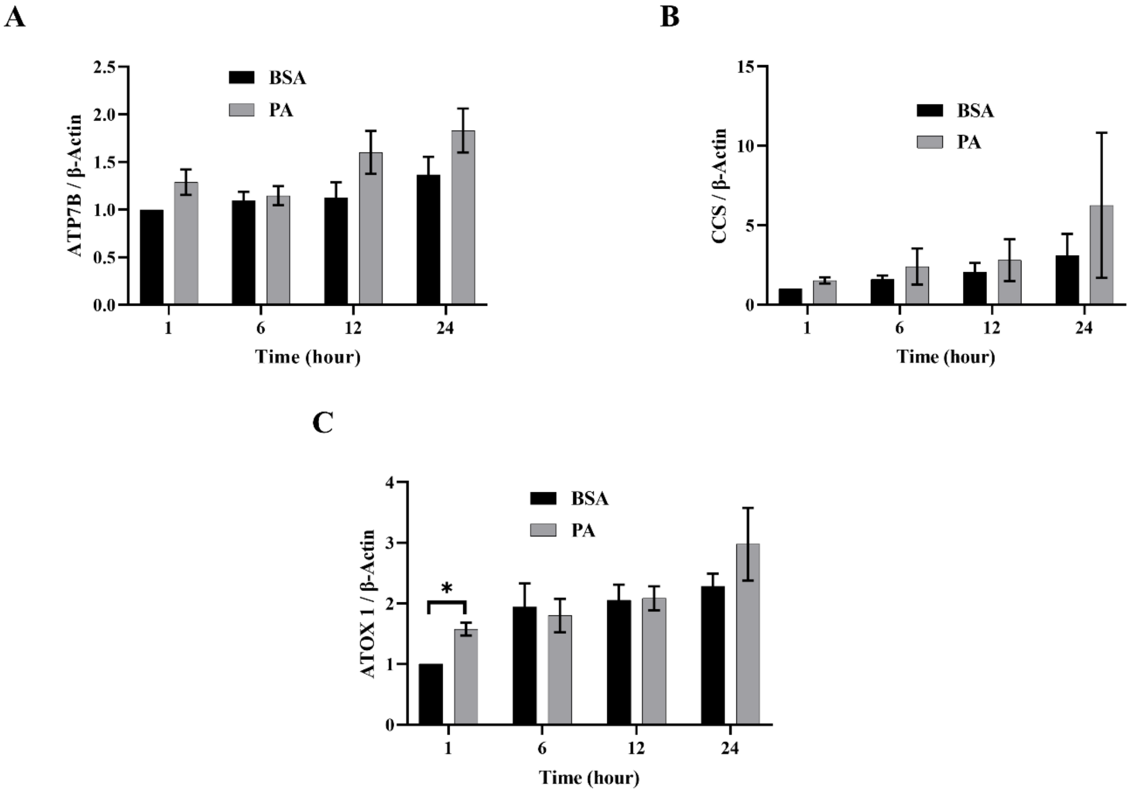

Supplemental Figure S1. Densitometry of representative blots are shown in Figure 1. Densitometry analysis of the Western blots of A) ATP7B (n = 7), B) CCS (n = 5), and C) ATOX1 (n=5). Mean ± SEM are shown. Unpaired Student's *t*-test, *p<0.05 was used to assess significance.

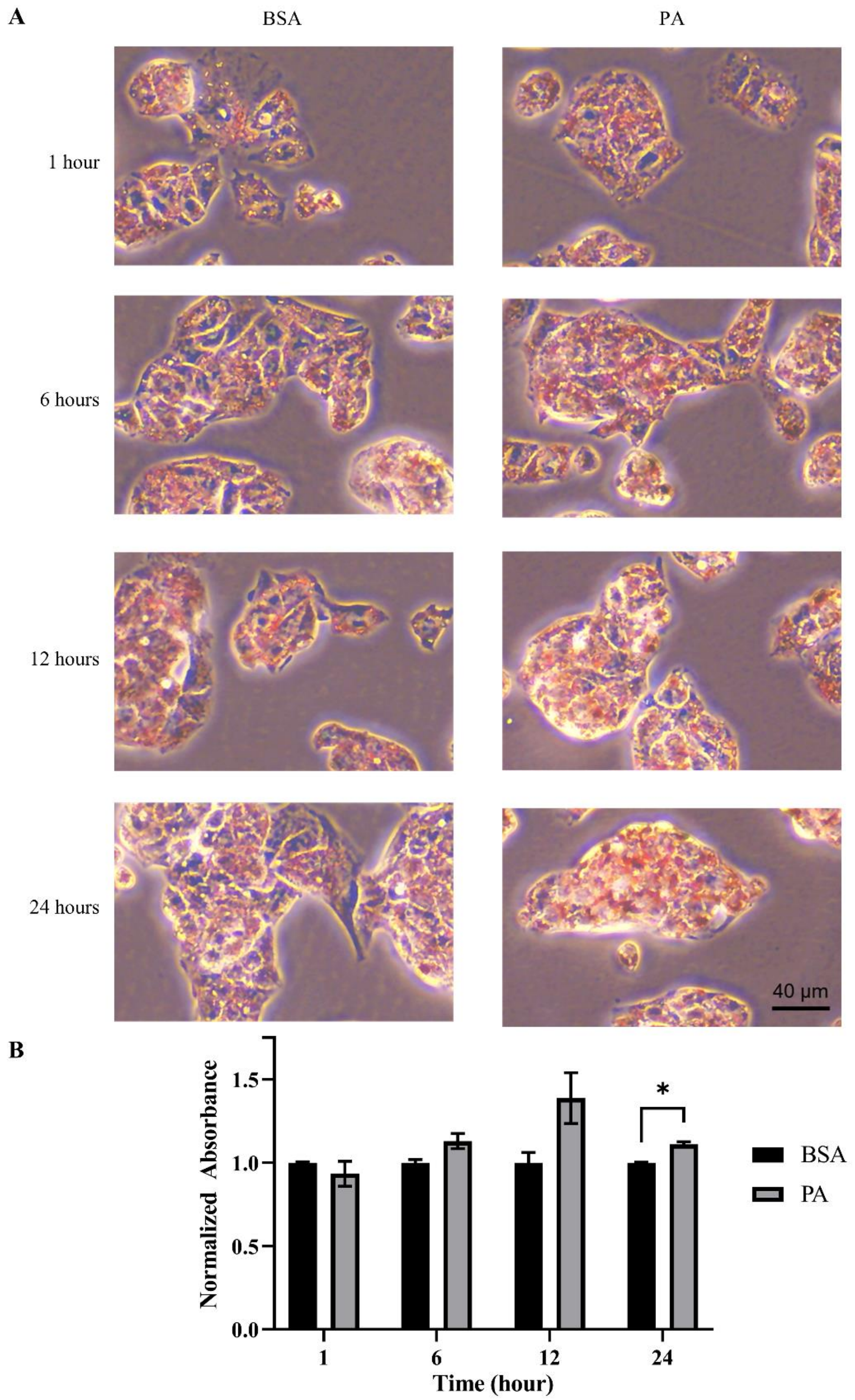

Supplemental Figure S2. PA induces fat accumulation in HepG2 cells. Oil O Red staining of HepG2 cells demonstrating intracellular fat accumulation in cells stimulated with 250 µM PA for 1, 6, 12, and 24 hours. A) Representative images of HepG2 cells are shown. B) Eluted Oil O Red dye from stained cells was measured at 540 nm to assess relative levels of fat accumulation within the cell (n =

4). Absorbance was normalized to BSA control at each time point. Mean ± SEM are shown. Unpaired Student’s *t*-test, *p<0.05 was used to assess significance.

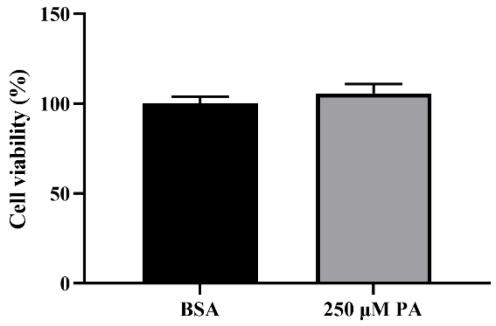

Supplemental Figure S3. Fat accumulation in HepG2 cells does not impact cell viability. HepG2 cells were stimulated for 24 hours with 250 µM PA. Cell viability was assessed by MTS assay (n = 4). Mean ± SEM are shown. Unpaired Student’s *t*-test, **p<0.01 was used to assess significance.

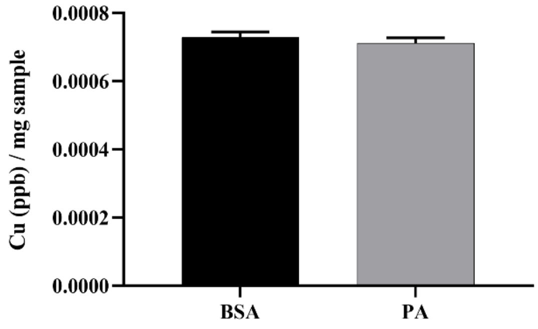

Supplemental Figure S4. No difference is observed in the copper levels between the BSA and PA stimulation media. ICP-MS analysis of stimulation media demonstrates no significant differences in the copper levels of the control (BSA) and PA stimulation media (n = 5). Mean ± SEM are shown.

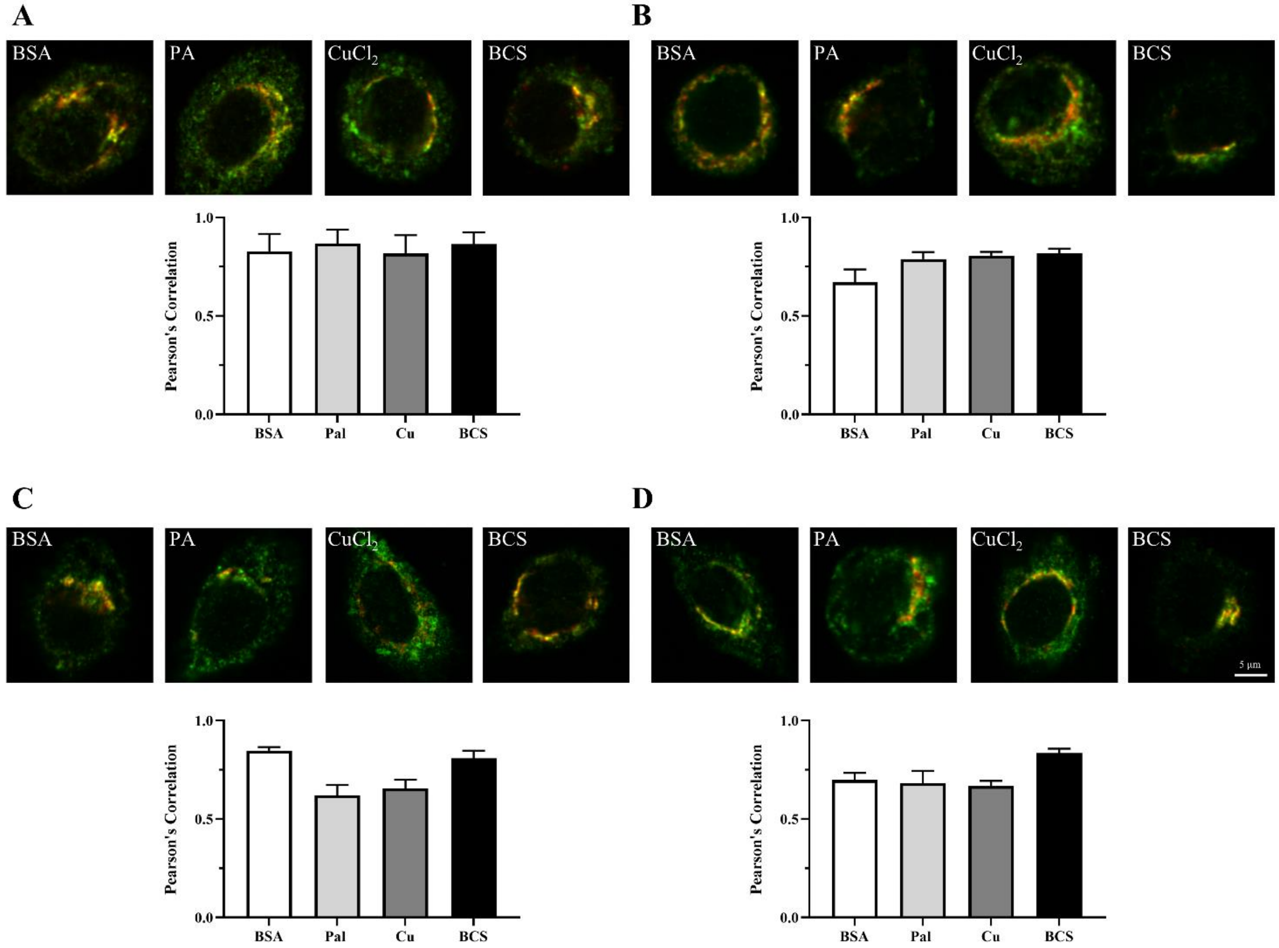

Supplemental Figure S5. Palmitate stimulation induces localization of ATP7B similar to copper copper deficient and copper overload states and early and late time points respectively. HepG2 cells were stimulated with PA, BSA, 200 µM $CuCl_2$ to induce a state of copper overload, or 200 µM BCS to induce a state of copper deficiency for A) 1, B) 6, C) 12, and D) 24 hours. Cells were fixed and immunostained, and immunofluorescence imaging was used to capture the subcellular localization of ATP7B (green) and TGN46 (red). Cells were imaged by laser scanning confocal microscopy with a 60x oil immersion lens. Colocalization is observed by the overlap of signals of ATP7B and TGN46 (yellow). Bar graphs demonstrate Pearson correlation coefficient used to assess colocalization of ATP7B and TGN 46 (n = 9). Mean ± SEM are shown.

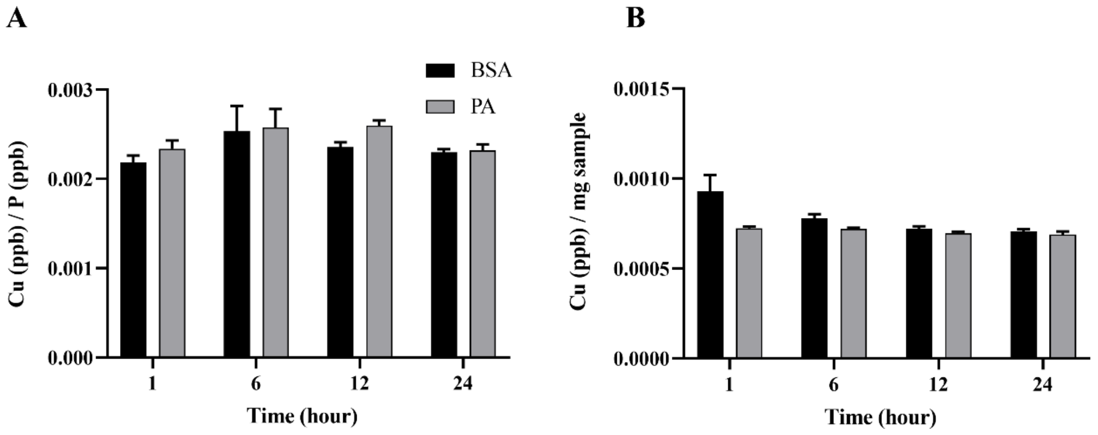

Supplemental Figure S6. PA stimulations do not induce significant changes in intra- or extracellular copper concentrations. Total copper levels analyzed by ICP-MS. A) Copper levels of whole cell pellets are expressed as copper concentration (ppb) over phosphorus concentration (ppb) (n = 5). B) Extracellular copper levels from the cell culture media are expressed as copper concentration (ppb) over mg sample (n = 5). Mean ± SEM are shown.

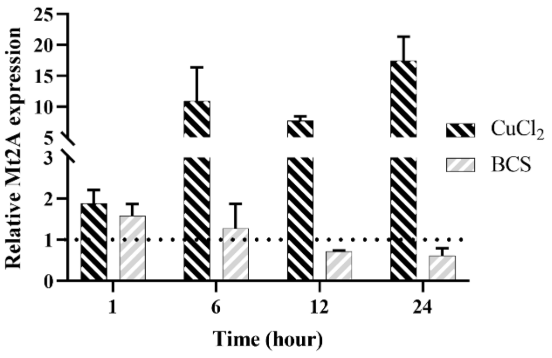

Supplemental Figure S7. Mt2A gene expression is sensitive to copper-modulating treatments. Gene expression analysis of Mt2A of cells treated with 200 µM $CuCl_2$ or 200 µM BCS relative to BSA-stimulated cells (n = 3) with normalization to β-Actin as the housekeeping gene. Mean ± SEM are shown.

Gene	Sequence (5’-3’)
B-actin Forward	GGACGACATGGAGAAAATCTGGCA
B-actin Reverse	GTAGATGGGCACAGTGTGGGTG
TBP Forward	CAGCCTTCCACCTTATGCTC
TBP Reverse	TGCTGCTGTCTTTGTTGCTC
CCS Forward	GACCCTCTGCACGTTGGAGTT
CCS Reverse	GTGGTGTGTACCAAGACCATCTG
ATP7B Forward	CTCATTAAAGCTACCCACG
ATP7B Reverse	GACAAAATATCCACTAAACCG
SOD1 Forward	ACTCTCAGGAGACCATTGCATCA
SOD1 Reverse	TCCTGTCTTTGTACTTTCTTCATTTCC
SCO2 Forward	TCCATTGCCATCTACCTGCTCAAC
SCO2 Reverse	TCAAGACAGGACACTGCGGAA
COX17 Forward	AGGAGAAGAAGCCGCTGAAG
COX17 Reverse	GGCCTCAATTAGATGTCCACAGT
Mt2A Forward	AAAGGGGCGTCGGACAAGT
Mt2A Reverse	TAGCAAACGGTCACGGTCAG
ATOX1 Forward	CTCTCGGGTCCTCAATAAGC
ATOX1 Reverse	GTTGCAAGCAGAGTGTCCAT
COMMD1 Forward	GCTGGAGAGTTGATGGCAAGTC
COMMD1 Reverse	GACCTCATCAAATTCCAAACACAG
Cp Forward	TCCCTGGAACATACCAAACC
Cp Reverse	CCAATTTATTTCATTCAGCCGA

Supplemental Table S1. RT-qPCR primer sequences
